# Copper-Induced Membrane Depolarizations Involve the Induction of Mosaic TRP Channels, Which Activate VDCC Leading to Calcium Increases in *Ulva compressa*

**DOI:** 10.3389/fpls.2016.00754

**Published:** 2016-06-14

**Authors:** Melissa Gómez, Alberto González, Claudio A. Sáez, Alejandra Moenne

**Affiliations:** ^1^Laboratory of Marine Biotechnology, Faculty of Chemistry and Biology, University of Santiago of ChileSantiago, Chile; ^2^Laboratory of Coastal Toxicology, Center of Advanced Studies, University of Playa Ancha Viña del Mar, Chile

**Keywords:** calcium, copper, marine alga, TRP channels, *Ulva compressa*, voltage-dependent calcium channels

## Abstract

The marine macroalga *Ulva compressa* (Chlorophyceae) is a cosmopolitan species, tolerant to heavy metals, in particular to copper. *U. compressa* was cultivated with 10 μM copper for 12 h and membrane depolarization events were detected. First, seven depolarization events occurred at 4, 8, 12–13, 80, and 86 min, and at 5 and 9 h of copper exposure. Second, bathocuproine sulphonate, a specific copper-chelating compound, was added before incorporating copper to the culture medium. Copper-induced depolarizations were inhibited by bathocuproine at 4, 8, 12–13, 80, and 86 min, but not at 5 and 9 h, indicating that initial events are due to copper ions entry. Third, specific inhibitors of human TRPA1, C4, C5, M8, and V1corresponding to HC030031, ML204, SKF96363, M8B, and capsazepin, respectively, were used to analyze whether copper-induced depolarizations were due to activation of transient receptor potentials (TRPs). Inhibitor effects indicate that the seven depolarizations involved the activation of functional mosaic TRPs that displayed properties similar to human TRPA, C, M, and/or V. Finally, inhibition of copper-induced depolarizations using specific TRP inhibitors suppressed calcium increases at 2, 3, and 12 h due to activation of voltage-dependent calcium channels (VDCCs). Thus, copper induces seven depolarization events that involve activation of mosaic TRPs which, in turn, activates VDCC leading to calcium increases at 2, 3, and 12 h in *U. compressa*.

## Introduction

Transient receptor potential (TRP) channels are ionotropic cation channels present in most, if not all, excitable and non-excitable mammalian, insect, and nematode cells (Madrid and Bacigalupo, 2015). TRPs are activated by a pletora of external stimuli such as injury, temperature, pH, osmolarity, pressure, pungent compounds, cytokines, prostaglandines, cannabinoids, diacylglycerol, inositol-phosphates, reactive oxygen species (ROS) and heavy metals, among others (Madrid and Bacigalupo, 2015). The first TRP channel was cloned from a mutant of *Drosophila melanogaster*, in which photoreceptors failed to sustain a light response (Montell and Rubin, 1989). Until now, 28 TRP channels have been identified in mammals and humans, which have been classified within six families, corresponding to TRPC (Canonical) with six members (TRPC1–TRPC6), TRPV (Vanilloid) with six members (TRPV1–TRPV6), TRPM (Melastatin) with eight members (TRPM1–TRPM8), TRPML (Mucolipin) with three members (TRPML1–TRPML3), TRPP (Polycystin) with three members (TRPP1–TRPP3), and TRPA (Ankyrin) with only one member, TRPA1 (Gees et al., 2010; Nilius and Owsianik, 2011; Madrid and Bacigalupo, 2015). In nematodes, an additional TRP has been identified corresponding to TRPN.

Transient receptor potential channels are constituted by six *trans*-membrane domains connected to intracellular N- and C-terminal domains, and the pore channel located between *trans*-membrane segments 5 and 6. Members of TRPC and TRPV families contain three to four ankyrin repeats in the N-terminal domain, whereas TRPA1 have 14 ankyrin repeats; TRPM family do not contain ankyrin domains (Gees et al., 2010; Nilius and Owsianik, 2011; Madrid and Bacigalupo, 2015). Ankyrin motifs participate in protein–protein interactions as well as in the binding of ligands such as ATP and calmodulins (CaMs; Gaudet, 2008).

Transient receptor potential channels such as TRPC1/5, TRPV5/6, and TRPM2 are highly permeable to calcium, whereas TRPV1 and TRPA1 are permeable to other cations such as sodium, potassium and magnesium (Gees et al., 2010; Zeng et al., 2012). In addition, it has been shown that metals ions such Mg^+2^, Mn^+2^, Ba^+2^, Zn^+2^, Ni^+2^, Co^+2^, and Sr^+2^ can permeate human TRP channels such as TRPA1, C5, and V1 (Bouron et al., 2015). On the other hand, human TRPs are also activated by heavy metals such as Pb^+2^ and Hg^+2^, as observed in TRPC5 (Sukumar and Beech, 2010; Xu et al., 2012), TRPA1, activated by Zn^+2^ (Hu et al., 2009), Cu^+2^ and Cd^+2^ (Gu and Lin, 2010), and TRPV1, stimulated by cations as Cu^+2^, Zn^+2^, Fe^+2^ (Riera et al., 2007; Cao et al., 2014), and Ni^+2^ (Luebbert et al., 2010). In addition, TRPs can be inhibited, as in the case of TRPM3, in response to Zn^+2^ (Yang et al., 2011), Hg^+2^, Fe^+2^, Se^+2^ (Zeng et al., 2012), and Cu^+2^ (Zeng et al., 2012; Yu et al., 2014). In addition, it has been shown that activation of human TRPs present in vascular smooth muscle cells leads to membrane depolarization, activating voltage-dependent calcium channels (VDCCs); the latter allows extracellular calcium entry which, in turn, promotes vasoconstriction (Brayden et al., 2008).

Regarding TRPs in algae little is known. Ten potential TRP channels have been identified in the genome of the green microalga *Chlamydomonas reinhardtii* (Arias-Darraz et al., 2015), and at least two of them have been identified to be functional (Fujiu et al., 2011; Arias-Darraz et al., 2015). More specifically, Cr-TRP11 located in the flagellum participates in the avoidance reaction of *C. reinhardtii* (Fujiu et al., 2011). On the other hand, molecular modeling of Cr-TRP1 showed that it is built as a mosaic TRP since it contains structural domains present in TRPM, N and C, and two ankyrin repeats in the N-terminal region (Arias-Darraz et al., 2015). Cr-TRP1 was cloned and expressed in human HEK-293T cells and whole cell patch-clamp studies demonstrated that it has better affinity for monovalent cation than for calcium, as human TRPM4 and M5 (Arias-Darraz et al., 2015). Moreover, Cr-TRP1 has been observed to get suppressed by BCTC, an inhibitor of TRPM8 and V1, with an IC_50_ of 1.03 μM (Arias-Darraz et al., 2015).

The marine alga *Ulva compressa* (Chlorophyceae) is a cosmopolitan species with enhanced tolerance to heavy metals, in particular to copper; indeed, it has been described in copper- polluted areas of northern Chile (Ratkevicius et al., 2003). It has been observed that *U. compressa* cultivated *in vitro* with a sub-lethal concentration of copper (10 μM) displayed intracellular calcium increases at 2, 3, and 12 h of exposure, which were due to calcium release from endoplasmic reticulum (ER; González et al., 2010a,b, 2012a). In addition, intracellular calcium release at 2, 3, and 12 h required extracellular calcium entry through VDCC, indicating that a calcium-induced calcium-release operates in response to copper excess (González et al., 2012b). It was recently shown that *U. compressa* contains functional TRPs that get activated in response to copper excess leading to extracellular calcium entry at 4, 9, and 12 min of exposure, allowing extracellular copper ions entry and inducing membrane depolarization events at 4, 8, and 12–13 min (Gómez et al., 2015). Copper-induced depolarization events were repressed by inhibitors of TRPA1, TRPC5, and TRPV1, suggesting that *U. compressa* TRPs may also correspond to functional mosaic TRPs, as Cr-TRP1 (Gómez et al., 2015). Considering that, in animals, activation of TRP channels leads to membrane depolarization which, sequentially, triggers activation of VDCC leading to extracellular calcium entry, it is possible that activation of *U. compressa* TRP channels and membrane depolarization events mediate the activation of VDCC and calcium increases at 1, 3, and 12 h of copper exposure.

In this work, we investigated the occurrence of additional membrane depolarization events that may happen until 12 h of copper exposure; the nature of TRPs involved in depolarization events that may occur; the involvement of protein kinases A and C in TRP-dependent membrane depolarization events; and the participation of TRPs in the activation of VDCC that may lead to intracellular calcium increases.

## Materials and Methods

### Algal and Seawater Sampling

*Ulva compressa* was collected in Cachagua (32° 34′S), a site with no history of metal pollution in central Chile (Ratkevicius et al., 2003); sampling occurred during autumn, winter and spring 2015. The algae were transported to the laboratory in sealed plastic bags inside a cooler at 4°C. Algal samples were rinsed three times with sterile filtered seawater and cleaned manually. Ultrasound was applied twice for 1 min using a Branson 3200 (Danbury, CT, USA) bath to aid removing epiphytic bacteria and organic debris. Seawater was obtained from the pristine site Quintay (33° 12′S) in central Chile; before the experiments, it was filtered through 0.45 and 0.2 μm pore size membrane and stored in the darkness at 4°C.

### Treatment with TRP Inhibitors

The inhibitors HC -030031, a specific inhibitor of TRPA1 (Eid et al., 2008), ML204, a specific inhibitor of TRPC4 (Miller et al., 2011), SKF96365, a specific inhibitor of TRPC5 (Merritt et al., 1990), M8B, a specific inhibitor of TRPM8 (Almeida et al., 2012), and capsazepin (CPZ), a specific inhibitor of TRPV1 (Maggi et al., 1993), were purchased form Sigma–Aldrich (St. Louis, MI, USA). Bathocuproine sulphonate, a specific copper-quelating compound (Mohindru et al., 1983); staurosporine, an inhibitor of PKA, PKC, PKG, and CaMKII (Meggio et al., 1995); KT5720, a specific inhibitor of PKA; and chelerythrine, a specific inhibitor of PKC (Herbert et al., 1990), were also purchased from Sigma–Aldrich (St. Louis, MI, USA).

To analyze copper-depolarization events, three laminae of the alga were incubated in 1 mL seawater and copper was added to a final nominal concentration of 10 μM; depolarization events were followed up until 12 h of copper exposure. To analyze whether depolarization events are due to copper ions entry, an additional treatment was conducted by incorporating bathocuproine sulphonate to a final concentration 500 μM before copper addition.

In a second experiment, to characterize the nature of TRPs leading to eventual depolarization events under short-term copper exposure, each specific inhibitor was added to final concentration of 20 nM in seawater containing three laminae of the algae, and then incubated for 40 min. Subsequently, 10 μM of the fluorophore DiOC2 were added and further incubated for 10 min. Finally, copper was added to a final concentration of 10 μM; the latter corresponded to time 0 min, and depolarization events were recorded for 1 h.

In a third experiment, to characterize TRPs involved in depolarization after 1 h and up to 12 h, 20 nM of each the specific inhibitors were added 40 min before the depolarization event occurred and the fluorophore 10 min before depolarization events. In a fourth experiment, to analyze the involvement of protein kinases in TRP activation, staurosporine, KT5720 and chelerythrine were used as described for the inhibitors in the second and third experiments, depending on the time the depolarization event occurred. In a fifth experiment, to analyze whether activation of TRPs are involved in VDCC activation, TRP inhibitors were added as described for the second and third experiments, and 20 μM of the fluorophore Fluo 3-AM was added 10 min before calcium increases are known to occur, at 2, 3, and 12 h of culture (see González et al., 2012b). All experiments described in this section were performed as three independent replicates.

### Detection of Membrane Depolarization Events

Detection of depolarization events was performed as described in detail by Gómez et al. (2015). DiOC2 (Molecular Probes, Invitrogen, Eugene, OR, USA) was added to 1 mL of seawater at a final concentration of 10 μM containing three laminae of *U. compressa* and incubated for 10 min at room temperature. The laminae were washed three times with filtered seawater to remove fluorophore excess. The green fluorescence of DiOC2 in each lamina was visualized by confocal microscopy using an Axiovert 100 confocal microscope (Carl Zeiss, Oberkochen, Germany), with an emission wavelength of 488 nM produced by an argon laser and with a filter of 505–530 nM. The intensity of green fluorescence and the red fluorescence of chloroplasts was quantified in each lamina using the confocal microscope LSM510 software. The fluorescence intensity in each sample was normalized using chloroplasts autofluorescence.

### Detection of Intracellular Calcium Increases

Detection of calcium was performed as described in detail by González et al. (2012b). Fluo-3AM at a final concentration of 20 μM was added to 1 mL of seawater containing 3 laminae of *U. compressa* and incubated for 10 min at room temperature. The laminae were washed three times in filtered seawater to remove fluorophore excess. The green fluorescence of Fluo 3 was visualized in each lamina by confocal microscopy using an Axiovert 100 confocal microscope (Carl Zeiss, Oberkochen, Germany), an emission wavelength of 488 nM produced by an argon laser and with a filter of 505–530 nM. The intensity of green fluorescence and the red fluorescence of chloroplasts was quantified in each lamina using the confocal microscope LSM510 software. The fluorescence intensity in each sample was normalized using chloroplasts autofluorescence.

### Statistical Analyses

To assess for significant differences, data were subject to one-way analysis of variance (ANOVA) and *post hoc* Tukey Test at 95% confidence interval, previous to verify requirements or normality and homogeneity of variance. Analyses were conducted on three independent replicates.

## Results

### Copper-Induced Depolarizations and Copper Ions Entry

**FIGURE 1:**
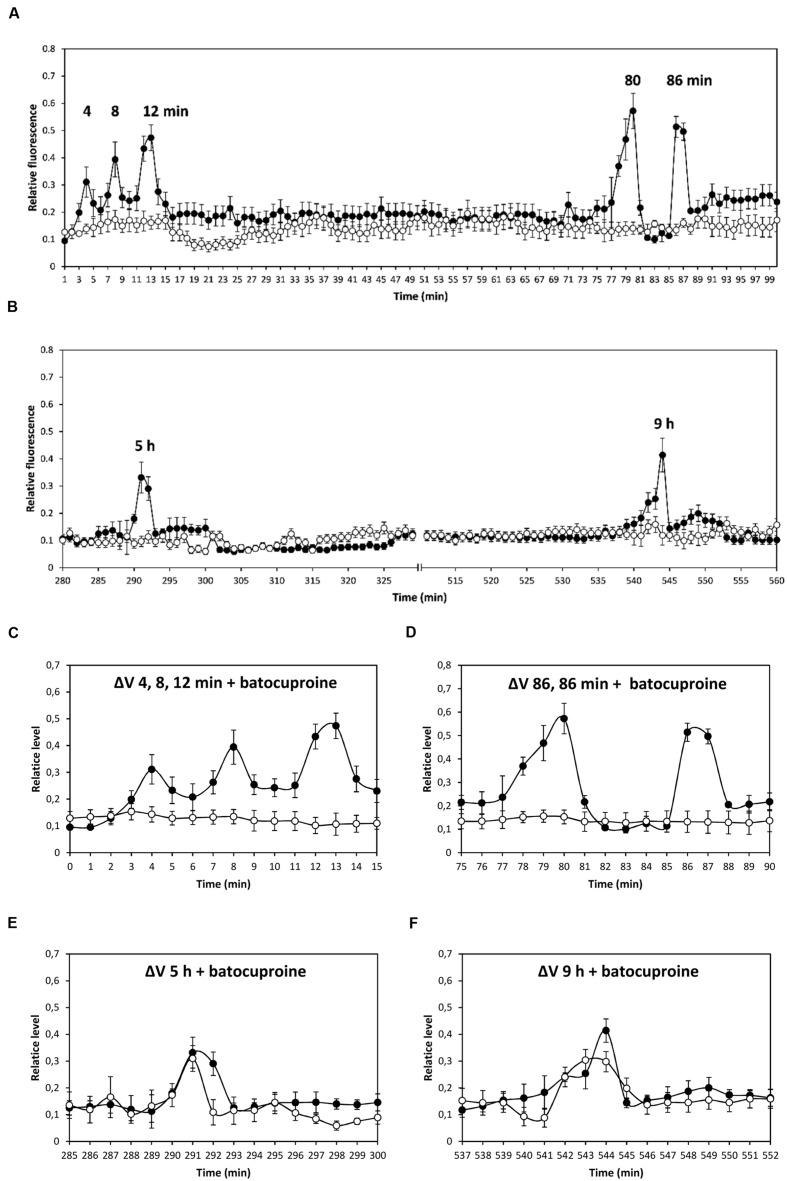
**Level of membrane depolarization (Δ*V*) in *Ulva compressa* cultivated with 10 μM copper from 0 to 99 min **(A)**, and from 250 to 600 min **(B)**.** Levels of membrane depolarization in *U. compressa* cultivated with 500 μM bathocuproine sulphonate (bathocuproine) and 10 μM copper between 0 to 15 min **(C)**, 75 to 90 min **(D)**, 285 to 300 min **(E),** and 537 to 552 min **(F)**. Levels of membrane depolarization in the alga cultivated with copper (black circles) and with bathocuproine and copper (open circles). Measurements represent mean values of three independent replicates ± SD.

The alga was cultivated in seawater with 10 μM copper for 12 h, and membrane depolarizations events were analyzed up to 99 min (**Figure 1A**), and between 250 and 600 min (**Figure 1B**). Depolarization events were observed at 4, 8, 12–13, 80, and 86 min (**Figure 1A**), as well as at 5 and 9 h (**Figure 1B**). No depolarization events were observed between 100 and 249 min (data not shown). To analyze whether depolarization events are due to copper ions entry, bathocuproine sulphonate, a specific copper-quelating compound, was added before copper addition. Bathocuproine inhibited copper-induced depolarizations at 4, 8, and 12–13 min (**Figure 1C**), as well as those at 80 and 86 min (**Figure 1D**); in contrast, depolarizations that occurred at 5 h (**Figure 1E**) and 9 h (**Figure 1F**) were not inhibited by bathocuproine. It is important to mention that calcium increases were not detected in parallel with depolarization events occurring between 80 min and 9 h (data not shown).

### Characterization of TRP Channels Involves in Copper-Induced Depolarizations

**FIGURE 2:**
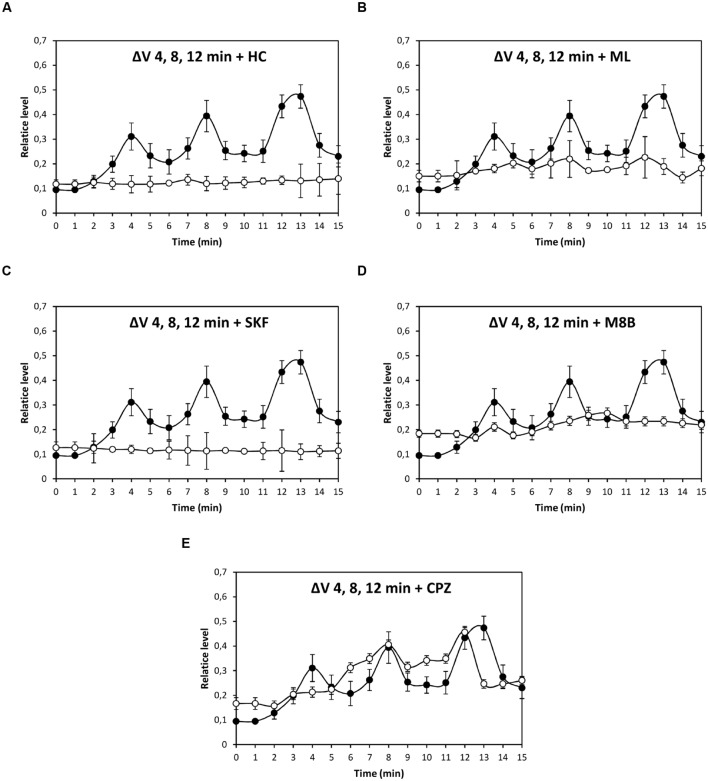
**Level of membrane depolarization (Δ*V*) in *U. compressa* cultivated with 10 μM copper up to 15 min and treated with of inhibitors HC030031 (HC, **A)**, ML204 (ML, **B)**, SKF96363 (SKF, **C)**, M8B (D), and capsazepin (CPZ, **E)** at a final concentration of 20 nM.** Levels of membrane depolarization in the algae cultivated with copper (black circles) and inhibitors and copper (open circles). Measurements represent mean values of three independent replicates ± SD.

In order to characterize the nature of TRP channels involved in copper-induced depolarizations, specific inhibitors of TRPA1, C4, C5, M8, and V1 corresponding to HC030031 (HC), ML204 (ML), SKF96363 (SKF), M8B, and CPZ, respectively, were used (**Figure 2**). Depolarization at 4 min was significantly inhibited by HC, ML, SKF, M8B, but not by CPZ (**Figure 2A**); depolarization events at 8 and 12–13 min were significantly inhibited by HC, ML, SKF, M8B, and CPZ (**Figures 2B,C**).

**FIGURE 3:**
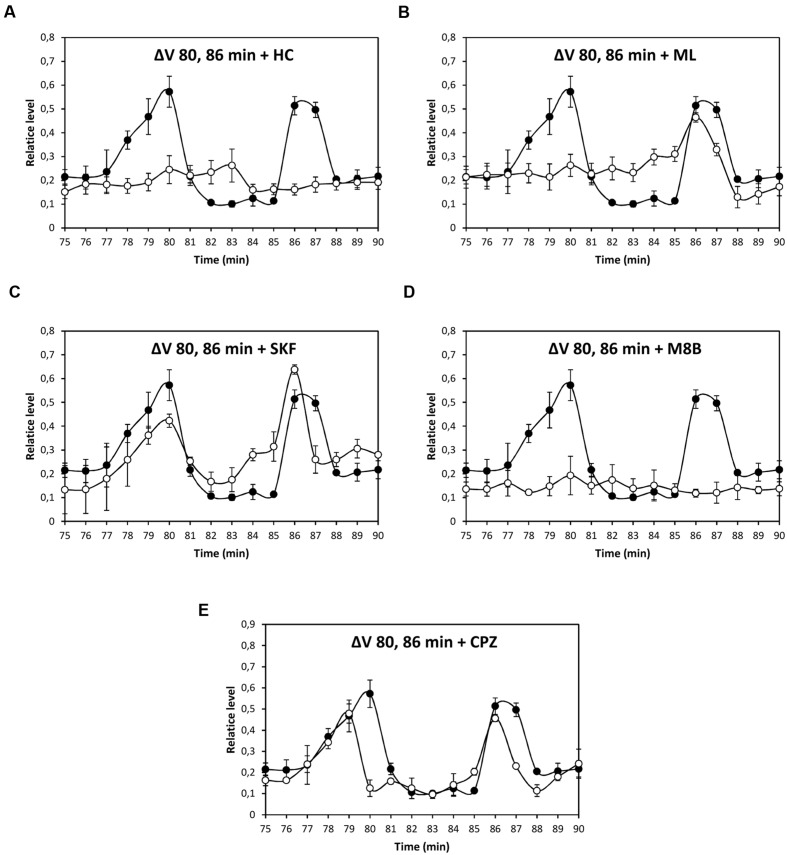
**Level of membrane depolarization (Δ*V*) in *U. compressa* cultivated with 10 μM copper up to 90 min and treated with inhibitors HC030031 (HC, **A)**, ML204 (ML, **B)**, SKF96363 (SKF, **C)**, M8B (D), and CPZ **(E)** at final concentration of 20 nM.** Levels of membrane depolarization in the algae cultivated with copper (black circles) and inhibitors and copper (open circles). Measurements represent mean values of three independent replicates ± SD.

**FIGURE 4:**
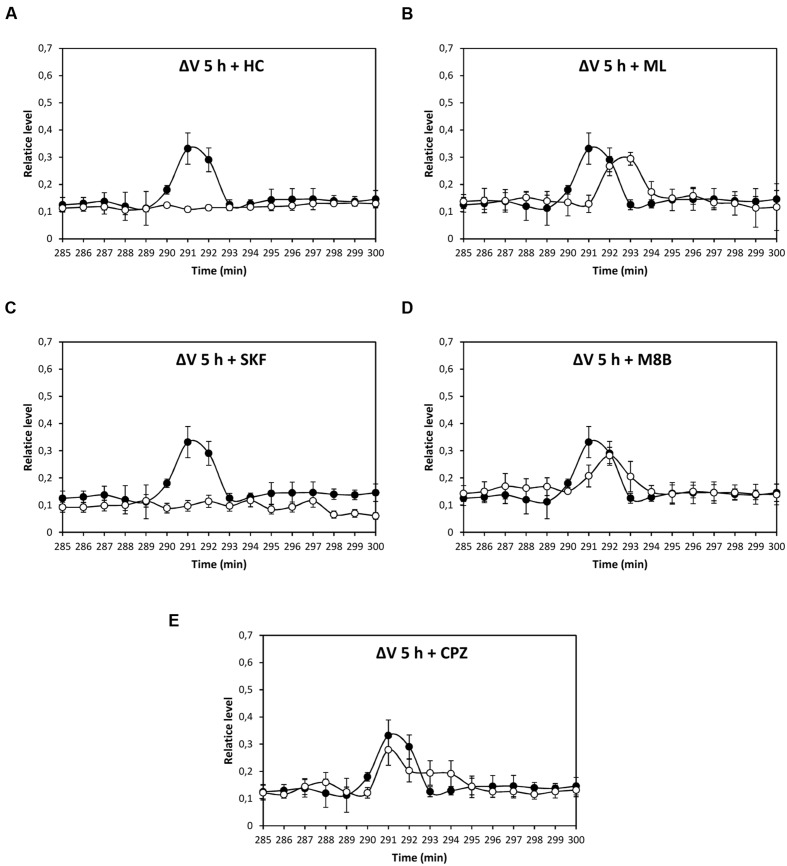
**Level of membrane depolarization (Δ*V*) in *U. compressa* cultivated with 10 μM copper up to to 300 min and treated with the inhibitors HC030031 (HC, **A)**, ML204 (ML, **B)**, SKF96363 (SKF, **C)**, M8B (D), and CPZ **(E)** at final concentration of 20 nM.** Levels of membrane depolarization in the algae cultivated with copper (black circles) and inhibitors and copper (open circles). Measurements represent mean values of three independent replicates ± SD.

**FIGURE 5:**
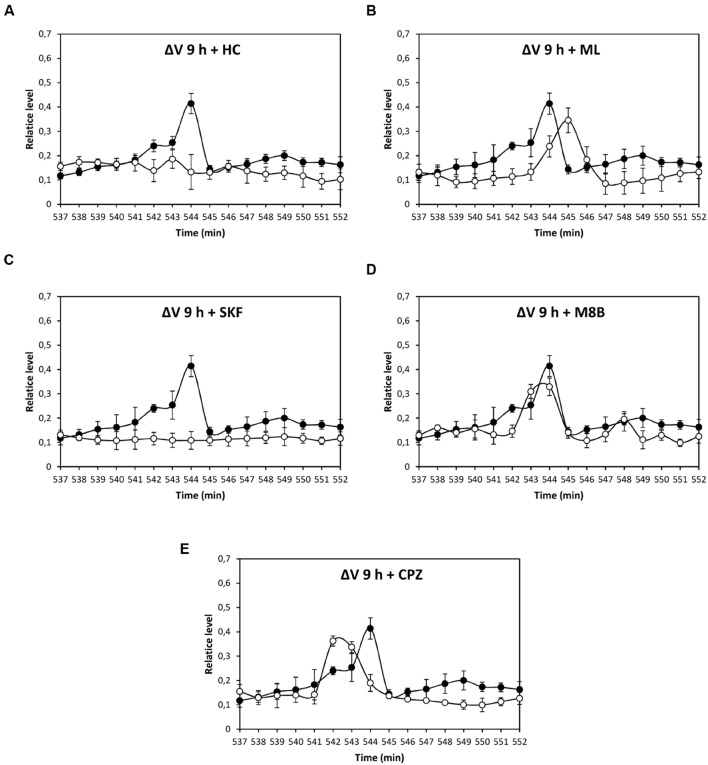
**Level of membrane depolarization (Δ*V*) in *U. compressa* cultivated with 10 μM copper up to 552 min and treated with the inhibitors HC030031 (HC, **A)**, ML204 (ML, **B)**, SKF96363 (SKF, **C)**, M8B (D), and CPZ **(E)** at final concentration of 20 nM.** Levels of membrane depolarization in the algae cultivated with copper (black circles) and inhibitors and copper (open circles). Measurements represent mean values of three independent replicates ± SD.

Depolarization event at 80 min was significantly inhibited by HC, ML, and M8B, but not by SKF and CPZ (**Figures 3A–E**), and depolarization at 86 min was significantly inhibited by HC and M8B, but not by ML, SKF, and CPZ (**Figures 3A–E**). The depolarization at 5 h was significantly inhibited by HC and SKF, but not by ML, M8B, and CPZ (**Figures 4A–E**), and the depolarization at 9 h was significantly inhibited by HC and SKF, but not by ML, M8B, and CPZ (**Figures 5A–E**). Thus, copper induced the activation of TRP channels displaying properties of human TRPs A, C, M and/or V.

### Involvement of Protein Kinases in Copper-Induced Depolarizations

In order to analyze the involvement of protein kinases in membrane depolarizations events, depolarizations were observed in the algae under copper and staurosporine (stau), an inhibitor of PKA, PKC, PKG, and CaMKII; copper and KT5720, a specific inhibitor of PKA; and copper and chelerythrine (chel), a specific inhibitor of PKC. Depolarization at 4 min was significantly inhibited by stau and KT5720, but not by chel; depolarization at 8 min was significantly inhibited by stau, KT5720 and chel; depolarization at 12–13 min was significantly inhibited by stau and K5720, but not by chel; depolarizations at 80 and 86 min were significantly inhibited by stau and chel, but not by K5720; depolarization at 5 h was significantly inhibited by stau, K5720, and chel; and depolarization at 9 h was not significantly inhibited by any of these inhibitors (**Table 1**).

**Table 1:** Events of copper-induced membrane depolarization in *Ulva compressa* eventually supressed by exposing the alga to the inhibitors staurosporine, KT5720, and chelerythrine at 20 nM final concentration.

Inhibitor	4 min	8 min	12 min	80 min	86 min	5 h	9 h
Staurosporine	+	+	+	+	+	+	-
KT5720	+	+	+	-	-	+	-
Chelerythrine	-	+	-	+	+	+	-


*Plus symbol (+) represents that the depolarization event was significantly inhibited, whereas minus symbol (-) indicates that the event was not significantly inhibited (95% confidence interval). Calculations were conducted on three independent replicates ± SD.*

### Copper-Induced Depolarizations are Involved in Activation of VDCC and Intracellular Calcium Increases

**FIGURE 6:**
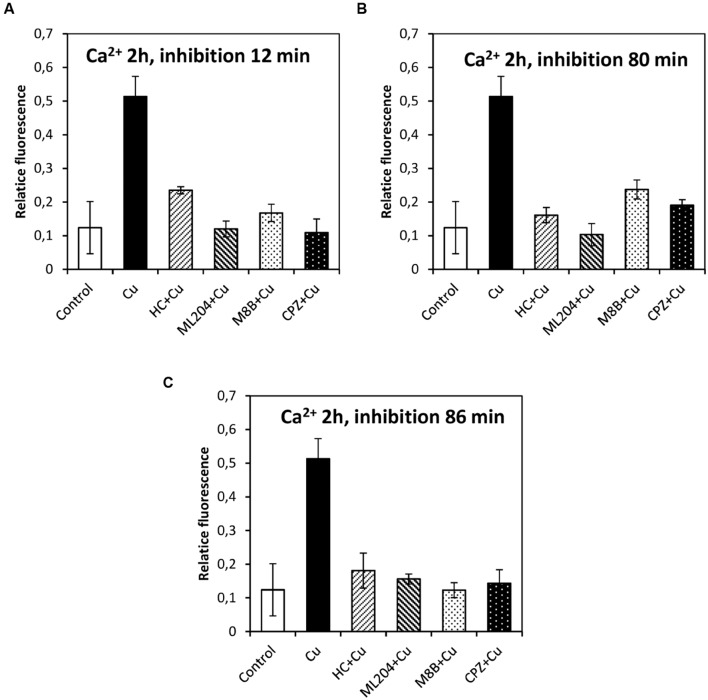
**Level of intracellular calcium in *U. compressa* known to occur at 2 h under 10 μM copper but treating the algae with the inhibitors HC030031 (HC), ML204 (ML), SKF96363 (SKF), M8B and CPZ for detected membrane depolarization events observed at 12 **(A)**, 80 **(B)** and 86 min **(C)**.** Bars represent mean values of three independent replicates ± SD.

**FIGURE 7:**
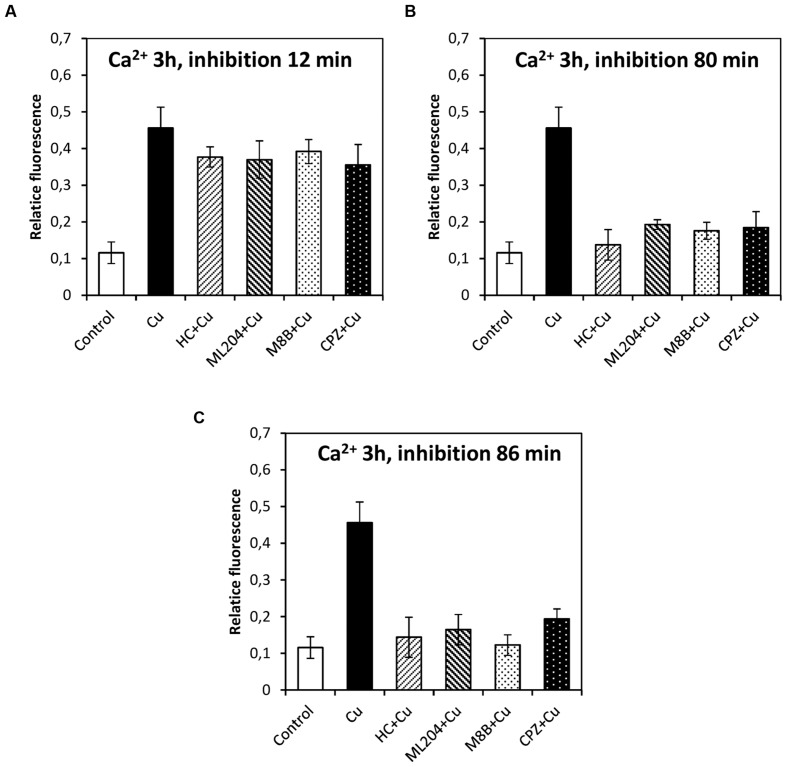
**Level of intracellular calcium in *U. compressa* known to occur at 3 h under 10 μM copper but treating the algae with the inhibitors HC030031 (HC), ML204 (ML), SKF96363 (SKF), M8B and CPZ for detected membrane depolarization events observed at 12 **(A)**, 80 **(B)** and 86 min **(C)**.** Bars represent mean values of three independent replicates ± SD.

**FIGURE 8:**
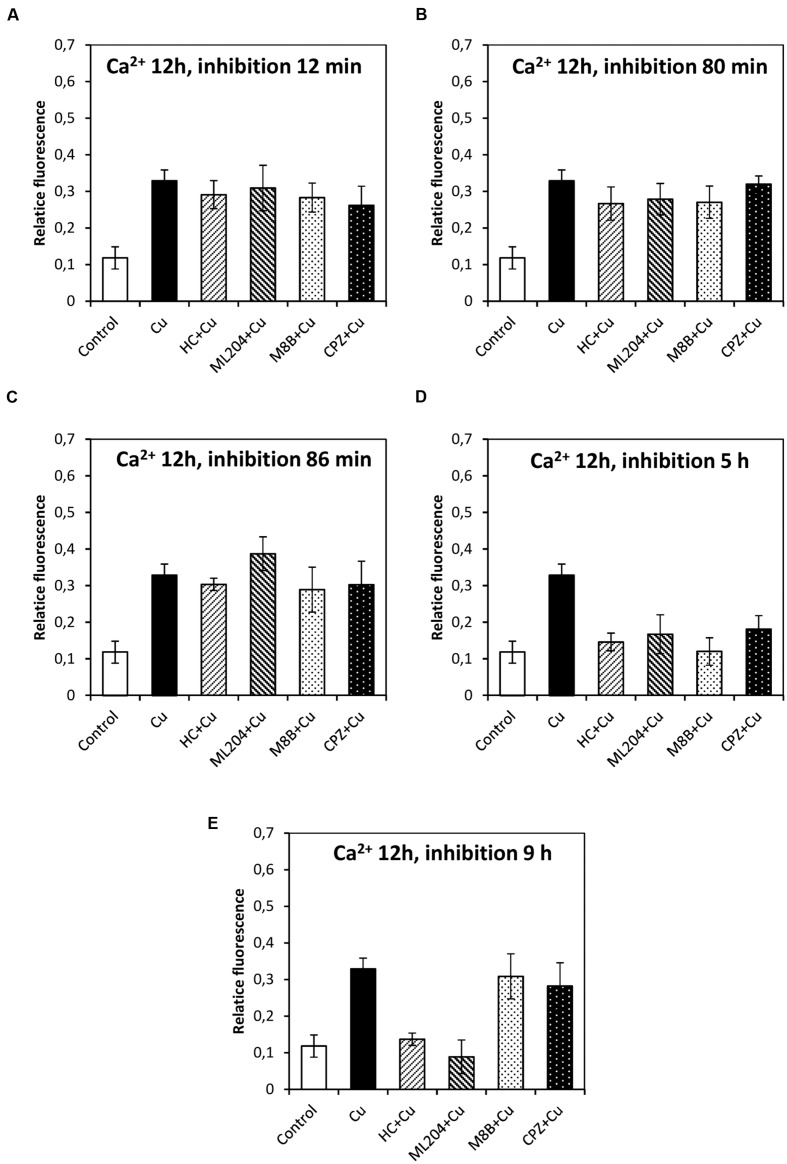
**Level of intracellular calcium in *U. compressa* known to occur at 12 h under 10 μM copper but treating the algae with the inhibitors HC030031 (HC), ML204 (ML), SKF96363 (SKF), M8B and CPZ for detected membrane depolarization events observed at 12 **(A)**, 80 **(B)**, and 86 min **(C)**, and at 5 **(D)** and 9 h **(E)**.** Bars represent mean values of three independent replicates ± SD.

In order to study whether the copper-induced depolarization events due to activation of TRPs are involved in the activation of VDCC which lead to intracellular calcium increase, the algae were incubated with specific inhibitors of TRPs (see past sections) at 12, 80, and 86 min, and 5 and 9 h. It is important to mention that increases in the levels of intracellular calcium were detected at 2, 3, and 12 h of copper exposure, in agreement with our previous investigations (González et al., 2012b). The increase in intracellular calcium at 2 h was significantly inhibited by HC, ML, M8B, and CPZ added at 12, 80, and 86 min (**Figures 6A–C**). The increase in intracellular calcium at 3 h was not significantly inhibited by HC, ML, M8B, and CPZ added at 12 min (**Figure 7A**), but it was significantly inhibited by HC, ML, M8B, and CPZ added at 80 min (**Figure 7B**) and at 86 min (**Figure 7C**). The increase in intracellular calcium observed at 12 h was not significantly inhibited by HC, ML, M8B, and CPZ added at 12 min (**Figure 8A**), at 80 min (**Figure 8B**), and 86 min (**Figure 8C**). Additionally, calcium increases at 12 h were significantly inhibited by HC, ML, M8B, and CPZ added at 5 h (**Figure 8D**), and only inhibited by HC and ML added at 9 h of copper exposure (**Figure 8E**).

## Discussion

In this work, we determined that the green macroalga *U. compressa* exposed to copper excess displayed seven membrane depolarization events until 12 h which occur at 4, 8, 12–13, 80, 86 min, and 5 and 9 h. In addition, the first five depolarization events were likely to be due to copper ions entry whereas the last two events did not appear to depend on copper ions entry. In this sense, it is important to point out that *U. compressa* TRPs are the only TRPs described to date that permeate copper ions, since human TRPs have been observed only to be permeable to metal ions such as Mg^+2^, Mn^+2^, Ba^+2^, Zn^+2^, Ni^+2^, Co^+2^, and Sr^+2^ through TRPA1, C5, and V1 (Bouron et al., 2015). The information suggests that *U. compressa* TRPs displays functional properties different with human TRPs. In addition, it was determined that copper-induced membrane depolarizations in *U. compressa* involved the activation of functional mosaic TRPs, if compared with human TRPs, since they displayed properties corresponding to TRPA, C, M, and V. Considering that human TRPV and C can be arranged as homo- or hetero-tetramers (Hofmann et al., 2002; Hellwig et al., 2005), it is possible that *U. compressa* TRPs may also correspond to a hetero-tetramers constituted by TRPA, C, M, and V subunits. However, it has been recently shown that a TRP1 in the green microalga *C. reinhardtii* is a mosaic TRP since it contains domains with high homology to TRPM, N, and C (Arias-Darraz et al., 2015). Moreover, it is possible that mosaic TRP subunits are able to form hetero-tetramer channels that allow copper ions entry after phosphorylation events (Gómez et al., 2015), which may explain functional differences among *U. compressa* and human TRP channels. In the future it would be interesting to clone and sequence *U. compressa* TRPs in order to determine whether TRPA, M, C, and V domains are present in these channels.

Furthermore, it was observed that copper-induced membrane depolarization events are dependent on the activation of PKA and/or PKC, which indicates that phosphorylation is required for the activation of TRPs in *U. compressa*. In this regard, it has been shown that human TRPV1 is activated by phosphorylation through PKC (Prenkumar and Ahern, 2000), TRPV4 activity is enhanced by phosphorylation through PKC (Fan et al., 2009), and TRPM4 and TRPC5 are activated by phosphorylation through PKC (Venkatachalam et al., 2003). Moreover, it has been identified that human TRPV1 is inhibited by phosphorylation through PKA (Mohapatra and Nau, 2003), TRPC3 is inhibited by phosphorylation mediated by PKC, and TRPC6 is inhibited by phosphorylate through PKG (Venkatachalam et al., 2003). Copper-induced depolarization events occurring at 8 min and 5 h required the activity of PKA and PKC and depolarizations at 4, 12–13, 80, and 86 min were not dependent on phosphorylation by PKA or PKC; however, were inhibited by staurosporine, which suggests that these depolarization events may require phosphorylation by PKG and/or CaMKII. The requirement of phosphorylation for the activation of *Ulva* TRPs is similar to what has been found for human TRPs.

**FIGURE 9:**
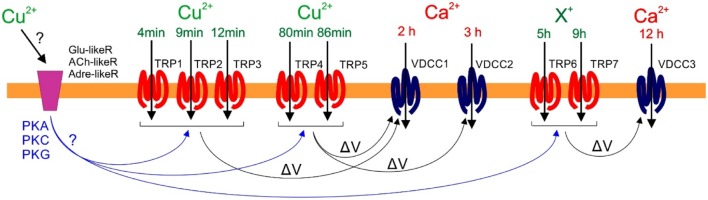
**Model of copper-induced transient receptor potential (TRP)-dependent depolarization events at 4, 8 12–13, 80, and 86 min, and 5 and 9 h leading to concomitant copper (Cu^+2^) or other cations (X^+^) entry, which participate in Voltage-Dependent Calcium channels (VDCC) activation and induce calcium ions (Ca^+2^) entry at 2, 3, and 12 h of 10 μM copper exposure.** It is proposed that that copper ions initially activate a glutamate-like receptor (Glu-likeR), an acetylcholine-like receptor (ACh-likeR), and/or an adrenalin-like receptor (Adre-likeR), which mediates the activation of proteins kinases PKA, PKC, or PKG; the latter induces the activation of TRPs at 4, 8 and 12–13, 80, and 86 min, and at 5 and 9 h of copper exposure leading to membrane depolarizations (Δ*V*) which, in turn, participate in the activation of VDCC at 2, 3, and 12 h facilitating calcium ions entry.

Finally, it was determined that copper-induced membrane depolarizations that involve the activation of TRP channels participate in the activation of VDCC leading to intracellular calcium increases at 2, 3, and 12 h. More precisely, depolarizations at 4, 8, 12–13, 80, and 86 min participate in calcium increases at 2 h, depolarizations at 80 and 86 min are involved in calcium increases at 3 h, and depolarizations at 5 and 9 h participate in calcium increases at 12 h (see a model in **Figure 9**). Therefore, there is a temporal coordination among copper-induced TRPs and VDCC activations. In humans, the involvement of TRPs in the activation of VDCC have been described, as in the case of TRPC3 and C6, process that allows extracellular calcium entry mediating the activation of VDCC and further calcium entry (Brayden et al., 2008). It is important to mention that the copper-induced activation of TRPs at 4, 8, and 12–13 min are not the initial events induced by copper excess, since these TRP-dependent depolarizations are inhibited by DNQX, an inhibitor of human glutamate (NMDA) receptors (Honoré et al., 1988), by propranolol, an inhibitor of β1/2 adrenaline/noradrenalin receptors (Bond, 1967), and curare, an inhibitor of nicotinic acetylcholine receptors (Pedersen and Cohen, 1990). Thus, our results suggests that it is possible that copper activates glutamate-like, adrenaline-like, and acetylcholine-like receptors in *U. compressa* (Gómez et al., unpublished); the latter leading to induction of protein kinases PKA, PKC, and/or PKG, which may participate in the activation of TRPs at 4, 8, and 12–13 min, 80 and 86 min, and 5 and 9 h (see model in **Figure 9**); although these hypotheses need to be further investigated.

## Conclusion

Copper induces the activation of TRP channels leading to membrane depolarizations at 4, 8, 12–13, 80, and 86 min, as well as at 5 and 9 h, which participates in the activation of VDCC at 2, 3, and 12 h, facilitating subsequent intracellular calcium increases. In addition, the functional properties of *U. compressa* TRPs suggest that they are built as a mosaic of TRPs, as they display properties of human TRPA, C, M, and V.

## Author Contributions

MG was involved in experimental design, conducted experiments and analyses, and interpretation of data. AG aid conducting experiments and analyses. CS was involved in data interpretation and manuscript writing up. AM supervised all stages of the investigation, interpreted results and manuscript writing up.

## Conflict of Interest Statement

The authors declare that the research was conducted in the absence of any commercial or financial relationships that could be construed as a potential conflict of interest.
